# Clinical efficacy on glycemic control and safety of mesenchymal stem cells in patients with diabetes mellitus: Systematic review and meta-analysis of RCT data

**DOI:** 10.1371/journal.pone.0247662

**Published:** 2021-03-11

**Authors:** Jingjing He, Desheng Kong, Zhifen Yang, Ruiyun Guo, Asiamah Ernest Amponsah, Baofeng Feng, Xiaolin Zhang, Wei Zhang, Aijing Liu, Jun Ma, Timothy O’Brien, Huixian Cui

**Affiliations:** 1 Hebei Medical University-National University of Ireland Galway Stem Cell Research Center, Hebei Medical University, Shijiazhuang, Hebei Province, China; 2 Hebei Research Center for Stem Cell Medical Translational Engineering, Shijiazhuang, Hebei Province, China; 3 The Fourth Hospital of Hebei Medical University, Shijiazhuang, Hebei Province, China; 4 Department of Epidemiology and Health Statistics, School of Public Health, Hebei Medical University, Shijiazhuang, Hebei Province, China; 5 Second Affiliated Hospital of Hebei Medical University, Shijiazhuang, Hebei Province, China; 6 Human Anatomy Department, Hebei Medical University, Shijiazhuang, Hebei Province, China; 7 Regenerative Medicine Institute, School of Medicine, National University of Ireland Galway, Galway, Ireland; Centro Cardiologico Monzino, ITALY

## Abstract

**Background:**

Diabetes mellitus as a chronic metabolic disease is threatening human health seriously. Although numerous clinical trials have been registered for the treatment of diabetes with stem cells, no articles have been published to summarize the efficacy and safety of mesenchymal stem cells (MSCs) in randomized controlled trials (RCTs).

**Methods and findings:**

The aim of this study was to systematically review the evidence from RCTs and, where possible, conduct meta-analyses to provide a reliable numerical summary and the most comprehensive assessment of therapeutic efficacy and safety with MSCs in diabetes. PubMed, Web of Science, Ovid, the Cochrane Library and CNKI were searched. The retrieval time was from establishment of these databases to January 4, 2020. Seven RCTs were eligible for analysis, including 413 participants. Meta-analysis results showed that there were no significant differences in the reduction of fasting plasma glucose (FPG) compared to the baseline [mean difference (MD) = -1.05, 95% confidence interval (CI) (-2.26,0.16), P<0.01, I^2^ = 94%] and the control group [MD = -0.62, 95%CI (-1.46,0.23), P<0.01, I^2^ = 87%]. The MSCs treatment group showed a significant decrease in hemoglobin (Hb) A1c [random-effects, MD = -1.32, 95%CI (-2.06, -0.57), P<0.01, I^2^ = 90%] after treatment. Additionally, HbA1c reduced more significantly in MSC treatment group than in control group [random-effects, MD = -0.87, 95%CI (-1.53, -0.22), P<0.01, I^2^ = 82%] at the end of follow-up. However, as for fasting C-peptide levels, the estimated pooled MD showed that there was no significant increase [MD = -0.07, 95%CI (-0.30, 0.16), P<0.01, I^2^ = 94%] in MSCs treatment group compared with that in control group. Notably, there was no significant difference in the incidence of adverse events between MSCs treatment group and control group [relative risk (RR) = 0.98, 95%CI (0.72, 1.32), P = 0.02, I^2^ = 70%]. The most commonly observed adverse reaction in the MSC treatment group was hypoglycemia (29.95%).

**Conclusions:**

This meta-analysis revealed MSCs therapy may be an effective and safe intervention in subjects with diabetes. However, due to the limited studies, a number of high-quality as well as large-scale RCTs should be performed to confirm these conclusions.

## Introduction

Diabetes mellitus (DM) is a chronic metabolic disease associated with major morbidity and mortality DM can be classified as type 1 diabetes (T1DM) when the pancreas fails to produce sufficient insulin due to autoimmune beta-cell destruction and type 2 diabetes (T2DM) when there is insufficient production of insulin and/or insulin resistance. Data from the International Diabetes Federation shows that the prevalence of diabetes among adults worldwide is 463 million and many of these patients are from China and India [1]. Among adults in China, the estimated prevalence of diabetes was 10.9%, and that for prediabetes was 35.7% [2]. There are many reasons for the increased prevalence of DM, including unhealthy lifestyles of diet and lack of exercise, an ageing population, genetic predisposition, and obesity [3–5]. Chronic hyperglycaemia as occur in DM leads to microvascular and macrovascular complications. The escalating number of diabetic patients and their complications have resulted in a higher mortality and heavy economic burdens on global care systems. Currently, conventional therapeutic regimens for diabetes which beyond diet and exercise include daily oral hypoglycemic agents and insulin injections are used to control high blood glucose. However, the conventional therapeutic regimens but they do not always cannot accurately control the dose of insulin efficiently regulate insulin levels and thus, may cause serious hypoglycemia, resulting in poor compliance, with a resultant poor attainment of target glycemic level. In the USA, only 14% of patients reach targets for glycemic control, lipids, blood pressure and smoking cessation. Despite many advanced research on the pathogenesis and pathobiology of diabetes, there remains an urgent need for more effective therapeutics to prevent and reverse this serious metabolic condition.

In the last decade, a growing number of animal experiments [6, 7] and clinical findings [8, 9] have suggested that MSC-based therapeutic intervention is an emerging and promising therapeutic modality for the treatment of diabetes due to the pleiotropic properties and low immunogenicity. Numerous clinical trials have been registered for the treatment of diabetes with MSCs, however, given the ethical issues and the complexity of the translational pathway, only a few have been published. Moreover, even with the few published studies, their findings were inconsistent. Some studies reported that the stem cell therapy for T1DM increased C-peptide levels and decreased glycosylated hemoglobin (HbA1c) [10, 11], but Carlsson et al. reported no significant difference in HbA1c or C-peptide levels after treatment with bone marrow-derived MSCs in T1DM [12]. As for T2DM, El-Badawy et al. found that stem cell therapy can improve C-peptide levels [10]. In addition, Zhang et al. also came to the same conclusions [11]. However, Rahim et al. showed that stem cell therapy decreases C-peptide in patients with T2DM [13]. The previous meta-analyses analyzed data from both RCTs and non-RCTs. However, due to selection bias and confounding factors, data from non-RCT may not be reliable in the evaluation of the efficacy of treatments. Therefore, we used data from RCTs to ascertain the efficacy and safety of MSC treatment in DM. In brief, our meta-analysis aims to critically evaluate and make the best use of clinical data on the efficacy and safety of MSC therapy for diabetes in RCTs. This study may help in the design of future clinical trials, and provide evidence for guidelines concerning the use of MSC therapy in DM.

## Research design and methods

### Eligibility criteria

Inclusion criteria included any age and sex of people diagnosed with DM [14], without any additional complications, and the trials compared MSCs with placebo or MSCs as an adjunct treatment to insulin. We excluded duplicate publications, non-human studies, reviews and comments, conferences and case reports, non-RCTs, and articles not reporting outcomes of interest or completed data. Included and excluded studies were screened following the Preferred Reporting Items for Systematic Reviews and Meta-Analyses (PRISMA) flow diagram [15].

### Outcomes

1. **Primary outcomes** Primary outcomes were changes in FPG, HbA1c, and fasting C-peptide between baseline and after therapy.
2. **Secondary outcomes** Secondary outcomes were adverse events including hypoglycemia, abnormal amylase, ketoacidosis.

### Searches strategy

We searched PubMed, Web of Science, Ovid, the Cochrane Library and CNKI databases using the following key words: (“mesenchymal stem cells, mesenchymal stromal cells, Wharton’s Jelly cells, progenitor cells, bone marrow” or “MSCs”) AND (“diabetes mellitus” or “hyperglycemia”) AND (“English language” OR “Chinese language”). The retrieval time was from establishment of these databases to January 4, 2020. The detailed search strategies are listed in Table 1.

**Table 1:** Search strategy.

Data source	Search terms
**PubMed**	#1 diabetes [MeSH Terms]
	#2 hyperglycemia [MeSH Terms]
	#3 mesenchymal stem cell [MeSH Terms]
	#4 mesenchymal stromal cell [MeSH Terms]
	#5 bone marrow stromal cell [MeSH Terms]
	#6 bone marrow derived stem cell [MeSH Terms]
	#7 mesenchymal progenitor cell [MeSH Terms]
	#8 Wharton’s Jelly cells [MeSH Terms]
	#9 #1 OR #2
	#10 #3 OR #4 OR #5 OR #6 OR #7 OR #8
	#11 #9 AND #10 AND "english"[Language]
**Web of science**	#1 TI = diabetes
	#2 TI = hyperglycemia
	#3 TI = mesenchymal stem cell
	#4 TI = mesenchymal stromal cell
	#5 TI = mesenchymal progenitor cell
	#6 TI = bone marrow stromal cell
	#7 TI = bone marrow derived stem cell
	#8 TI = Wharton’s Jelly cells
	#9 #1 OR #2
	#10 #3 OR #4 OR #5 OR #6 OR #7 OR #8
	#11 #9 AND #10
	#12 (#11) *AND* LANGUAGE: (English)
**Ovid**	#1 diabetes.m_titl
	#2 mesenchymal stem cell.m_titl
	#3 mesenchymal stromal cell.m_titl
	#4 mesenchymal progenitor cell.m_titl
	#5 bone marrow stromal cell.m_titl
	#6 bone marrow derived stem cell.m_titl
	#7 Wharton’s Jelly cells.m_titl
	#8 #2 OR #3 OR #4 OR #5 OR #6 OR #7
	#9 #8 AND #1
	#10 limit 9 to english language
	#11 limit 10 to yr =“1860–2019”
**The Cochrane Library**	#1 (diabetes mellitus):ti, ab, kw OR (hyperglycemia):ti, ab, kw (Word variations have been searched)
	#2 (mesenchymal stem cell):ti, ab,kw
	#3 (mesenchymal stromal cell): ti, ab,kw OR (mesenchymal progenitor cell):ti, ab, kw OR (bone marrow stromal cell):ti, ab, kw OR (bone marrow derived stem cell):ti, ab, kw OR(Wharton’s Jelly Cells):ti, ab, kw (Word variations have been searched)
	#4 #3 OR #2
	#5 #4 AND #1

### Trial selection and basic characteristics

Two researchers independently screened literatures and extracted data (He and Kong). Divergences were arbitrated and resolved by a third reviewer. For all eligible studies, we extracted information on first author name, published year, settings, study design, population characteristics, interventions, outcome of interest, duration of follow-up, adverse events, and risk classification (Table 2). When reports lacked information, we sent e-mails to contact corresponding authors. The units were transformed and unified based on related specifications. If the data were inconsistent from main body to tables, refer to the former.

**Table 2:** Baseline characteristics of the 7 included studies.

Studies	Nations	Diseases	No. of Patients (male)and Control	Mean Age (years)	BMI (kg/m2)	Cell Types	Cell Dose*Times	Delivery Methods	Follow-up Period	Outcomes
Bhansali et al. 2017 [21]	India	T2DM	10(8);10(6)	48.2;51.9	28.7;26.4	BM-MSCs	1×10^6^/kg*1	SPD artery Splenic artery	12m	①②③
Carlsson et al. 2015 [12]	Sweden	T1DM	9(8);9(5)	24;27	23.3;22.5	BM-MSCs	2.75×10^6^/kg*1	IV	12m	②③
Chen et al. 2016 [22]	China	T2DM	6(6);6(0)	57.5;57.5	23.35;13.63	UC-MSCs	1×10^6^/kg*4	Pancreatic artery +IV	24w	①②
Hu et al. 2013 [23]	China	T1DM	15(9);14(8)	17.6;18.2	20.9;21.3	UC-MSCs	2.6×10^7^*2	IV	24m	②
Hu et al. 2016 [24]	China	T2DM	31(17);30(16)	52.43;53.21	26.74;27.03	UC-MSCs	1×10^6^/kg*2	IV	36m	①②
Zhang et al. 2016 [25]	China	T2DM	16(9);17(7)	22.1;21.6	20.8;21.1	A-MSCs	1×10^7^/kg*1	IV	24m	①②③
Zhou et al. 2013 [26]	China	T2DM	120(60);120(61)	57.4;57.6	Unknown	BM-MSCs	1×10^7^/kg*3	Unknown	3w	①②③

Abbreviations: T1DM: type 1 diabetes mellitus, T2DM: type 1 diabetes mellitus, MBI: body mass index, BM-MSCs: bone marrow-mesenchymal stem cells, UC-MSCs: umbilical cord-mesenchymal stem cells, A-MSCs: amniotic-mesenchymal stem cells, SPD: superior pancreaticoduodenal artery, IV: intravenous. Outcomes: ①FPG, ②HbA1c, ③Fasting C-Peptide.

### Risk of bias (quality) assessment

Before statistical analysis, the Cochrane Risk of Bias tool was used to assess the quality of included studies [16]. The five domains we assessed were: random sequence generation, allocation concealment, blind, incomplete outcome data and selective reporting. The quality assessment of these studies is presented in Table 3.

**Table 3:** Evaluation of the methodological quality of the included studies.

Studies	Random Sequence Generation	Allocation Concealment	Blind	Incomplete Outcome Data	Selective Reporting	Other Sources of Bias
Bhansali et al. 2017 [21]	Random allocation software	Unclear	Double blind	Complete	Low risk	Unclear
Carlsson et al. 2015 [12]	Sealed envelopes	Yes	Open	Complete	Low risk	None
Chen et al. 2016 [22]	Randomization table	Unclear	Unclear	Complete	Low risk	Unclear
Hu et al. 2013 [23]	Randomized blocks	Unclear	Double blind	1 patient withdrawed	Low risk	None
Hu et al. 2016 [24]	Balanced permuted-block randomization method	Unclear	Unclear	Complete	Low risk	Unclear
Zhang et al. 2016 [25]	Unclear	Unclear	Unclear	Complete	Low risk	Unclear
Zhou et al. 2013 [26]	Unclear	Unclear	Unclear	Complete	Low risk	Unclear

### Data analysis

In this meta-analysis, we compared the MSC treatment groups from the selected trials with their respective control groups using Review Manager Version 5.3 software. The I^2^ statistic were calculated to assess heterogeneity among these trials. I^2^<50% was considered as a low level of heterogeneity, fixed-effects models were used; or random -effects models were used [17]. The MSCs medicinal effects were reflected by the mean MD with 95% CI, as well as P<0.05 was considered to be statistically significant. The adverse effect of treatment was summarized using RR.

## Results

### Trial selection and basic characteristics

**Fig 1:**
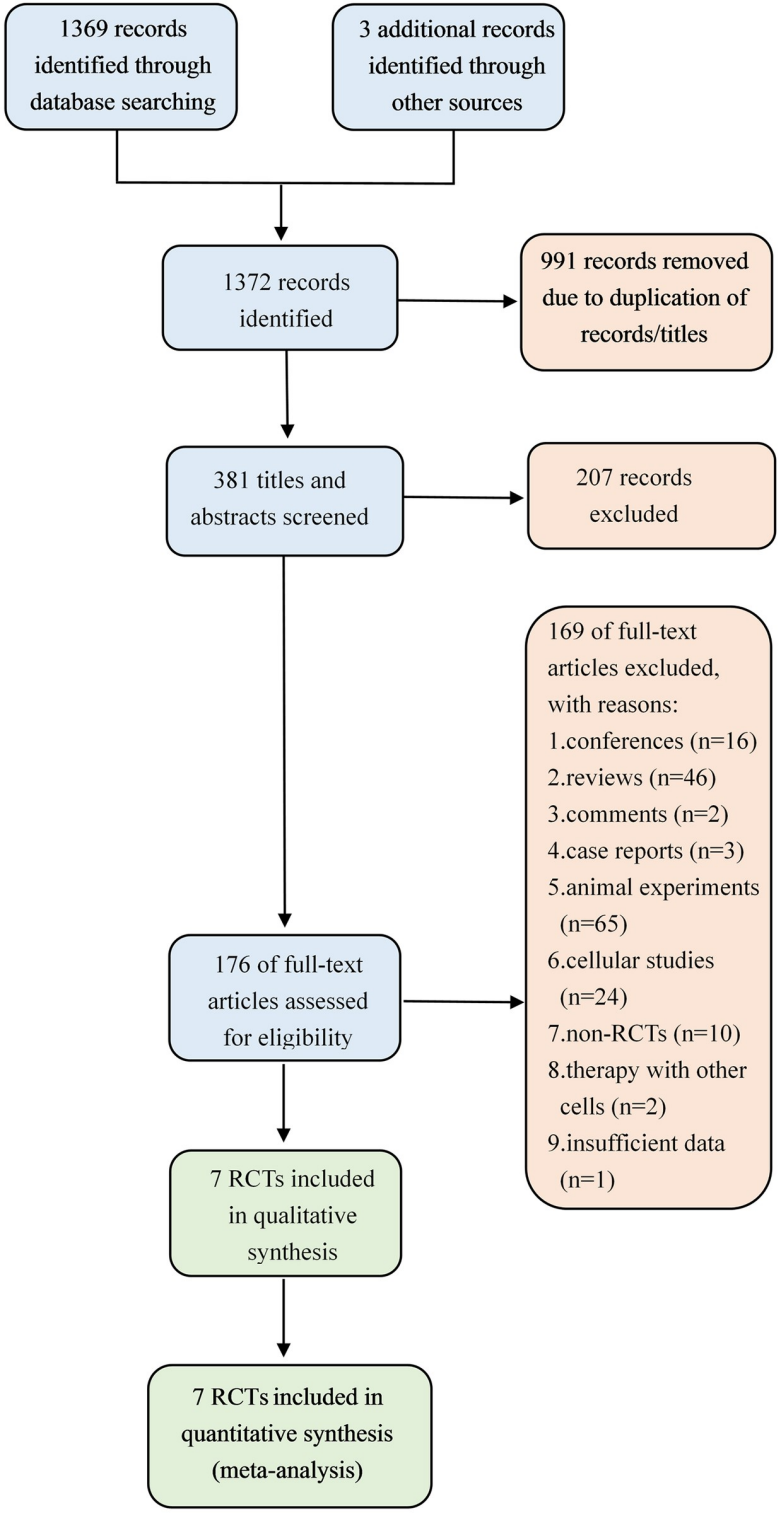
Flow diagram showing the study identification, screening, and inclusion process.

We initially retrieved 1,372 citations from selected databases and prior bibliographies. Of these, the majority were excluded. After 176 full-text articles were assessed, 169 studies were excluded because they were non-human clinical trials, non-RCTs, and combining many complications with type 2 diabetes (T2DM). Three studies [18–20] were subsequently excluded because they used other stem cells simultaneously and did not provide available data. Finally, a total of 7 RCTs were eligible for inclusion in the meta-analysis and were assessed for quality [12, 21–26]. A flow diagram showing the selection process of studies is summarized in Fig 1.

### Study characteristics

The baseline characteristics of the included trials are presented in Table 1. Overall, there were 413 participants (50.1% with MSCs; n = 207). Most of these trials were carried out in China, 1 was carried out in India and 1 was carried out in Sweden. The mean age of these subjects ranged from 17.6 to 57.6 years, and predominantly were male sex. However, other clinical information from the trials such as body weight, blood pressure, liver and renal function tests, fasting plasma insulin and insulin requirement were not collected because of insufficient data on some trials. Mainly three different types of MSCs involved in the eligible studies included bone marrow MSCs (BM-MSCs), Wharton’s jelly MSCs (WJ-MSCs), umbilical cord MSCs (UC-MSCs), and amniotic MSCs (AMSCs) with use of various cell doses, respectively. The number of stem cells transfused into patients in these studies was >1.0×10^7^/kg via intravenous or intra-arterial delivery. Of these, 2 studies reported data on MSCs and T1DM [12, 23], and 5 studies reported data on MSCs and T2DM [21, 22, 24, 25]. Based on initial hypoglycemic therapy, subjects from control arms were randomly assigned to receive a placebo [22–24] or a sham procedure [21] or neither [25]. At the beginning of therapy, only 2 studies included with HbA1c<7.5%. In addition, 2 patients from two different studies were withdrawn after enrolled: one was from MSCs treatment group [21] and the other from control group [23], respectively. Notably, 1 out of 7 studies [21] only reported median, maximum and minimum, and 2 reported mean and standard error of mean (SEM) [12, 23]. In the case of no result in contacting the original authors, median, range, SEM and sample size were used to estimate mean and standard deviation (SD) [27]. Different units were applied after unification for the same observed index.

### Glycemic efficacy outcomes

The mean values and SD of FPG, HbA1c, and fasting C-peptide levels before and after treatment, or between MSC therapy groups and control groups were compared.

#### 1 FPG

**Fig 2:**
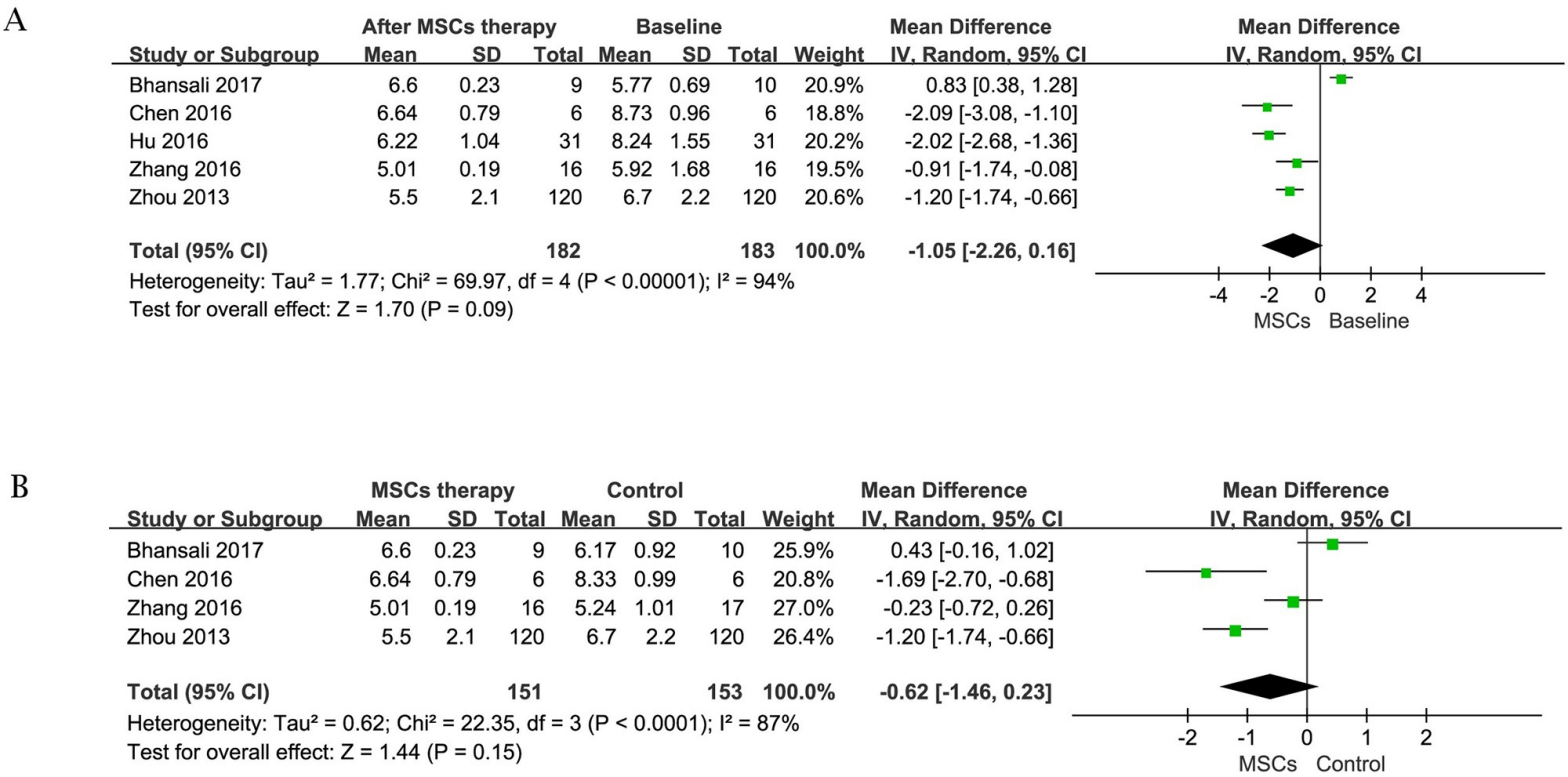
Forest plot for FPG (mmol/l). Comparison of FPG before MSCs therapy and after MSCs therapy (A); Comparison of FBG between the MSCs therapy group and control group (B).

FPG is widely used in the clinical diagnosis for DM as a vital metabolic parameter. Data on the change of FPG before and after MSCs treatment was available in 5 trials [21–26] containing 183 subjects at initial stage, including 1 patient lost to follow-up in MSCs therapy group after 6 months [21]. The efficacy of MSCs therapy in the estimated pooled MD showed no significant difference [random-effects, MD = -1.05, 95%CI (-2.26,0.16), P<0.01, I2 = 94%] in the reduction of FPG after cell treatment (Fig 2A).

**Fig 3:**
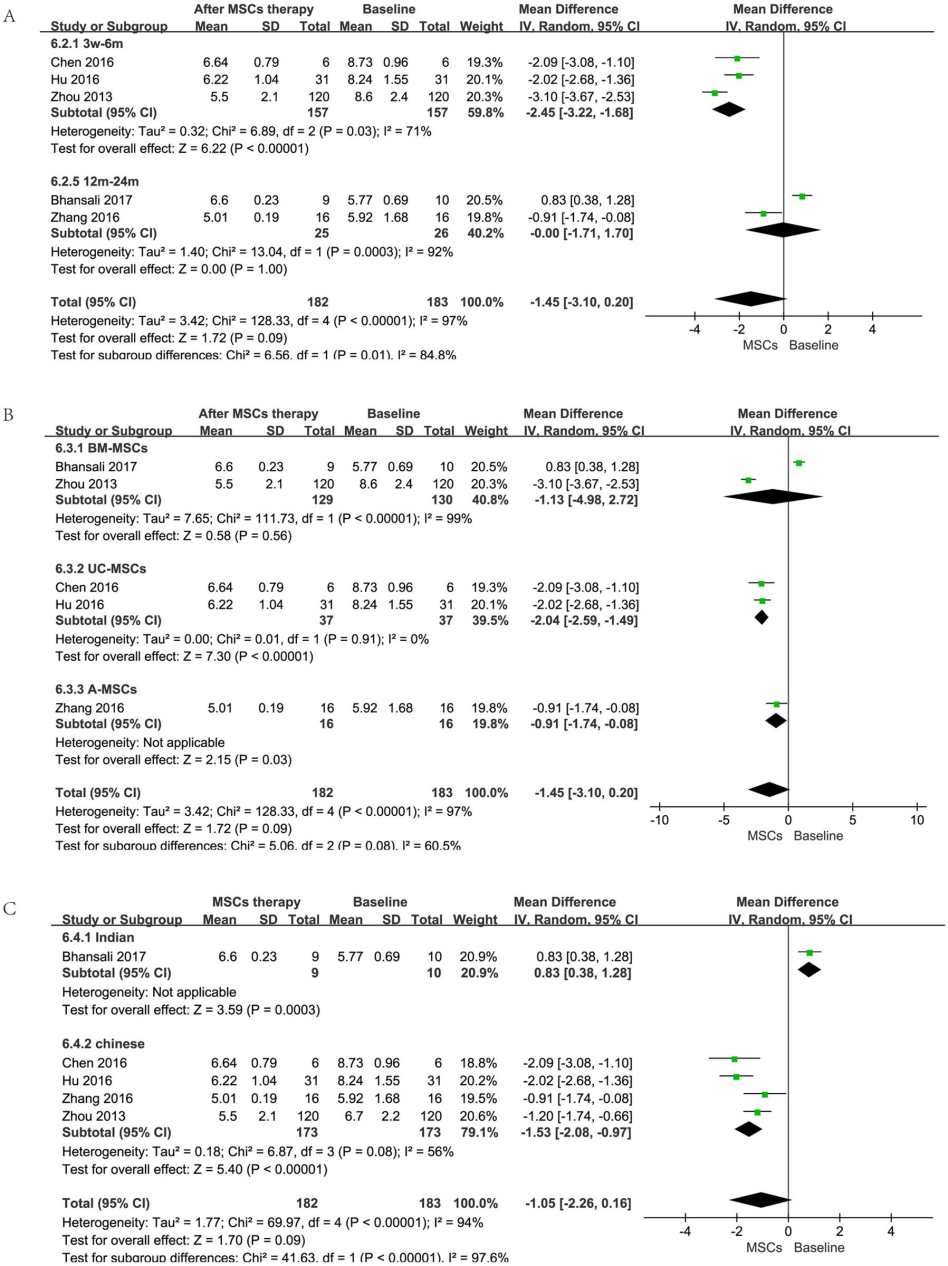
Forest plot for subgroup analysis of FPG (mmol/l). Comparison of FPG from different follow-up period before MSCs therapy and after MSCs therapy (A); Comparison of FPG from different cell types before MSCs and after MSCs therapy (B); Comparison of FPG from different nations before MSCs therapy and after MSCs therapy (C).

However, when these studies were removed in subgroup analysis according to time, cell types, and races of the primary outcome (FPG), results showed that the type of the infused cells did not affect the outcome; short-term (less than 6 months) follow-up displayed a better effect; studies in China showed much smaller heterogeneity than total and revealed statistically significant differences in FPG between MSCs therapy group and baseline (Fig 3). But for all this, more studies are needed to confirm these findings as these data are from a subgroup analysis. On the other hand, 4 trials [21, 22, 25, 26] (153 patients with MSCs therapy) allowed the change of FPG in MSC therapy group and control group at the end of follow-up to be evaluated. Compared with control group, there were no significant differences [random-effects, MD = -0.62, 95%CI (-1.46,0.23), P<0.01, I^2^ = 87%] in the reduction of FPG in MSC therapy group (Fig 2B).

#### 2 HbA1c

**Fig 4:**
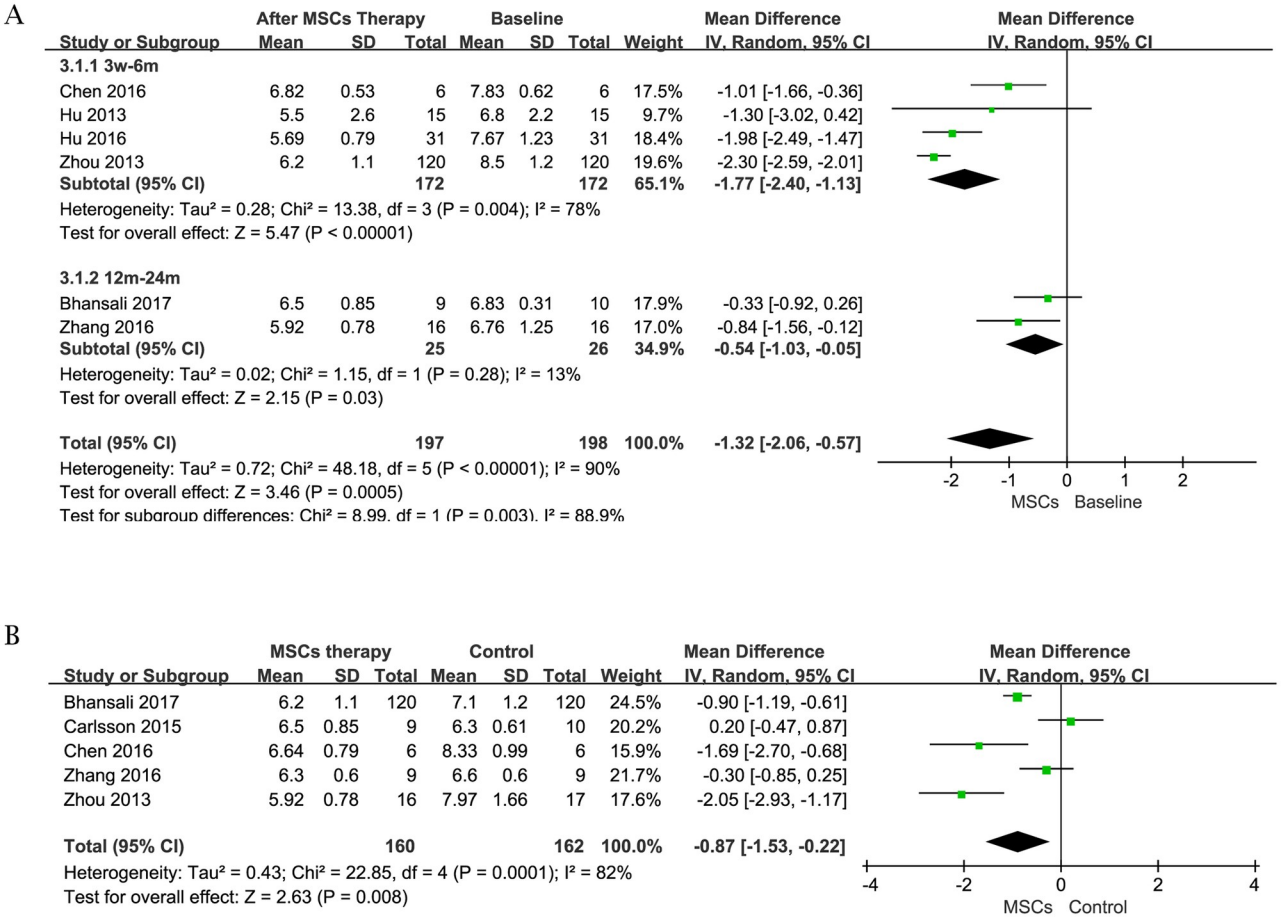
Forest plot for HbA1c (%). Comparison of HbA1c before MSCs therapy and after MSCs therapy (A); Comparison of HbA1c between the MSCs therapy group and control group (B).

We compared the change of HbA1c in the different stages before and after MSCs treatment to show the effect of the therapy. Information was available in 6 trials [21–26] containing 198 subjects at initial stage, including 1 patient lost to follow-up in MSCs therapy group after 6 months [21]. The estimated pooled MD showed significant reduction [random-effects, MD = -1.32, 95%CI (-2.06, -0.57), P<0.01, I^2^ = 90%] in HbA1c after MSCs treatment (Fig 4A). Meanwhile, 5 trials [21, 22, 25, 26] containing 322 subjects (160 patients with MSCs therapy) were analyzed to assess the change of HbA1c in MSCs therapy group and control group at the end of follow-up. Compared with the control, MSC treatment group showed a significant decrease [random-effects, MD = -0.87, 95%CI (-1.53, -0.22), P<0.01, I^2^ = 82%] in HbA1c (Fig 4B).

#### 3 Fasting C-peptide

**Fig 5:**
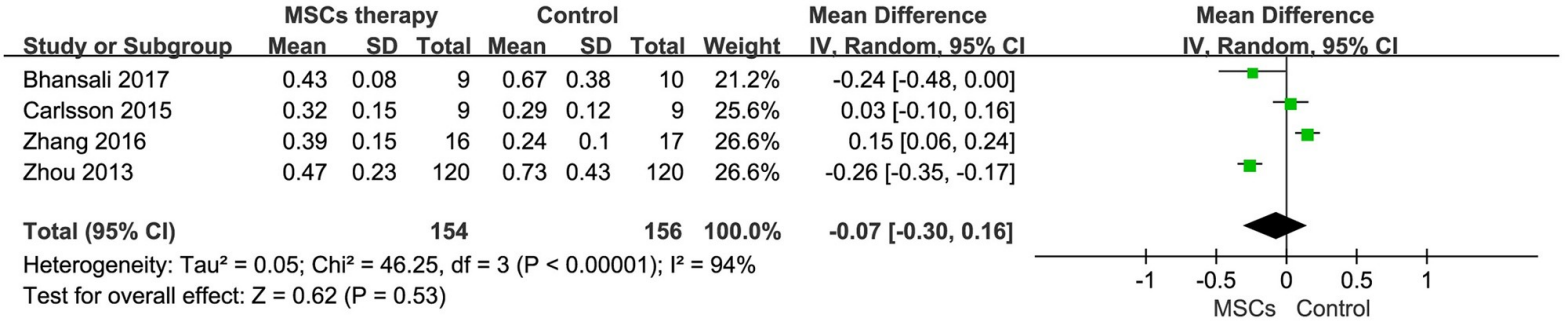
Forest plot for fasting C-peptide (nmol/L). Comparison of C-peptide level between the MSCs therapy group and control group.

Data on the fasting C-peptide levels that was available in 4 trials [12, 21, 25, 26]. The total sample size was 310, including 154 cases in MSCs treatment group and 156 cases in control group. Of these, Bhansali et al. and Zhou et al. ’s studies [21, 26] showed a reduction in fasting C-peptide levels after MSCs treatment at the end-point of follow-up. Carlsson et al. found fasting C-peptide levels were rescued at ten weeks in T1DM patients, but the effect of therapy diminished so quickly that no significant differences in level was observed at 12 months compared to the control [12]. However, Zhang et al. found that fasting C-peptide level significantly increased in MSCs treatment group after 24-month follow-up [25]. The estimated pooled MD for the 4 studies showed that there were no significant differences [MD = -0.07, 95%CI (-0.30, 0.16), P<0.01, I^2^ = 94%] in MSCs treatment group (Fig 5).

### Safety outcome

**Fig 6:**
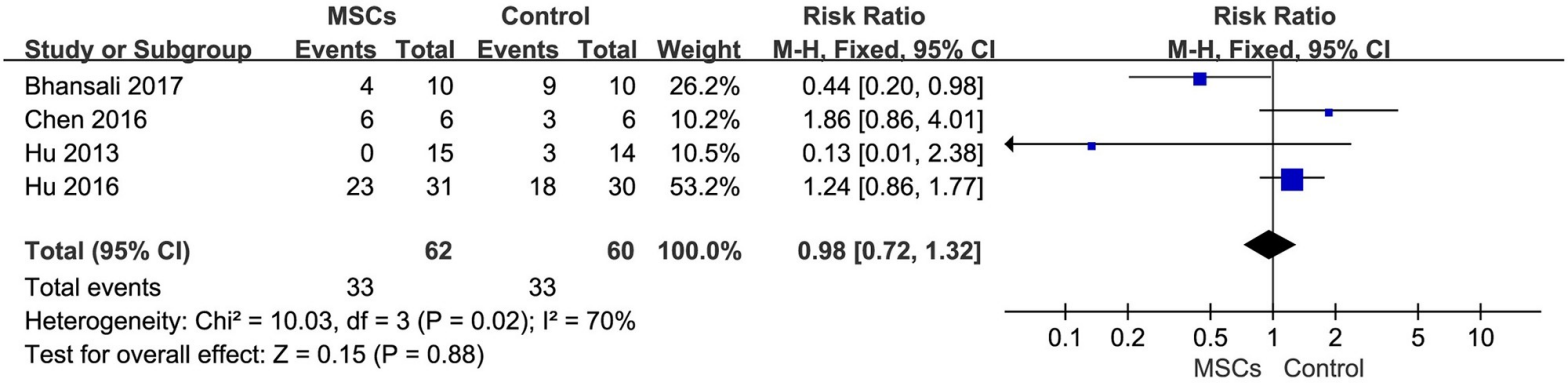
Forest plot of the incidence of adverse events in MSCs therapy group and control group.

A total of 6 trials and 66 adverse reactions were reported where 33 were from MSCs therapy group. Of these, no adverse event happened in 2 trials that were not shown in the forest plot. No subjects had a serious adverse event related to MSCs therapy in the included RCTs during the follow-up period. The side effects were mild and included hypoglycemia, abnormal amylase and ketoacidosis. There was no statistically significant increase in the incidence of adverse events between the two groups [RR = 0.98, 95%CI (0.72, 1.32), P = 0.02, I^2^ = 70%] (Fig 6).

The most commonly observed adverse reaction was hypoglycemia (29.95%) in the MSC therapy group. The heterogeneity was low, suggesting a satisfactory security.

## Discussion

The purpose of this meta-analysis was to evaluate the clinical data and obtain summary statistics for therapeutic interventions using MSCs in DM to improve glycemic control and to provide an evidence-based and cost-effective medical approach of this therapy. Several studies have evaluated the therapeutic potential of stem cell therapy in DM. However, these findings were inconsistent Our analysis shows that MSC infusion significantly improves HbA1c levels, although there were no significant differences in FPG and fasting C-peptide levels in patients with T1DM and T2DM. Moreover, there is no significant difference in the incidence of adverse effects between the two groups. To sum up, this meta-analysis suggests MSC-based stem cell therapy can improve glycemic control with a favorable safety profile and may become an alternative therapeutic regimen for diabetic patients.

DM is a heterogeneous disease and is characterized by hyperglycemia, oxidative stress, abnormal immune responses and chronic inflammation, which contribute to many related complications. Despite multifaceted improvements in self-management, synthetic drugs and insulin therapy, there appears to be evidence of a resurgence in diabetic complications in the last decade [28]. Meanwhile, diabetes remains the major cause of end-stage renal disease worldwide [29]. Therefore, intensified glycemic control including the use of insulin injections and new medications such as SGLT-2 inhibitors may not be adequate to normalize glycemic control and avoid complications.

Stem cells have properties of self-renewal, high proliferation potential and directional differentiation ability in certain conditions. While there is controversy surrounding the stem cell nature of MSCs, this cell type has become an attractive candidate for cell-based regenerative medicine with its multidirectional differentiation potential and immunomodulatory properties. Many of the therapeutic approaches being explored are based on the paracrine properties of these cells and particularly the immunomodulatory and anti-inflammatory effects. Moreover, MSCs can be harvested from a variety of sources including bone marrow, umbilical cord, Wharton’s Jelly, amnion, adipose tissues, and even urine in a relatively simple way. In particular, they are easily expanded in vitro and identified according to a detailed surface expression profile [30]. MSCs have been suggested as a therapy for diabetes mellitus in addition to being assessed for treatment of specific complications. In this meta-analysis we focused on the therapeutic use of MSCs in the treatment of diabetes per se and not its complications. Hence, we included the most widely studied MSCs derived from bone marrow, umbilical cord and amnion tissue in our meta-analysis. In recent years, a growing body of experimental and pre-clinical evidence has demonstrated the effectiveness of MSCs transplantation in DM. Si et al. found that BM-MSCs infusion ameliorated hyperglycemia in type 2 diabetic rats via increasing the expression of GLUT4 and insulin receptors to improve insulin sensitivity [31]. Besides, Pan et al. observed a noteworthy change in a type 2 diabetes macaque model that FPG levels were lower and the serum C-peptide levels were increased after MB-MSCs transplantation in contrast with control group [32]. These and other studies have now allowed study of the effects of MSC infusion on glycemic control in humans.

Similarly, some authors have demonstrated WJ-MSCs can differentiate into insulin-producing cells in vivo with immunomodulatory effects and repair the destroyed islets in diabetic rats and mice [33, 34]. However, these abilities remain controversial, since some researchers reported insulin production merely after a genetic manipulation or specific pro-differentiative factors [35–37]. On the other hand, an alternative microenvironment such as hyperglycemia and metabolic disturbance in diabetes could impact MSC biology and function, which may limit the therapeutic effects of MSCs on the engraftment [38, 39]. Apart from the stand-alone therapy with MSCs, MSCs and islet co-transplantation has also received more attention. In 2018, a study showed the ability of MSCs to inhibit the apoptotic pathway activation by endoplasmic reticulum stress in transplanted pancreatic islets in mice. In the same year, Wang et al. demonstrated that autologous BM-MSCs reduced FPG and improved the success rates of islet transplantation in patients with specific types of diabetes [40]. Taken together, although these papers confirmed the feasibility of MSCs for the treatment of diabetes, the majority of clinical trials had short follow-up or inadequate sample size. Now larger studies especially RCTs are necessary to validate their effectiveness and safety.

As far as we know, the present meta-analysis is the first attempt to systematically collect all RCTs and critically assess and quantify the efficacy and safety of MSC therapy for DM, both type 1 and 2. We noted that the previous 3 meta-analyses also retrieved the information about stem cell and DM (1 T2DM study, 2 T1DM+T2DM studies), the conclusions were not completely uniform [10, 11, 41]. Of these, Wang et al. observed a decrease in HbA1c and FPG, and an increase of C-peptide level after bone marrow mononuclear cells or peripheral blood mononuclear cells therapy in T2DM patients. Besides, El-Badawy et al. showed the mean HbA1c level was reduced and the mean C-peptide peak level was elevated after stem cells therapy in T1DM and T2DM patients [10]. Although Zhang et al. also found stem cell therapy improved HbA1c and C-peptide levels in T1DM and T2DM patients, they found there was no significant change in FPG levels for T2DM patients [11]. However, Carlsson et al. reported no significant difference in HbA1c or C-peptide levels after treatment with bone marrow-derived MSCs in T1DM [12]. The differences, by contrast, between this meta-analysis and aforementioned studies are more specific cellular types and more rigorous data extraction. We included the most widely studied MSCs for DM therapy and extremely strict data transformation according to original results. Therefore, we carried out the current study and systematically analyze FPG, HbA1c and Fasting C-peptide levels as well as adverse events after MSC therapy. Firstly, most of RCTs focus on T2DM, and MSCs as add-on treatment to insulin can decrease HbA1c levels. Secondly, MSC-based cell treatment was associated with follow-up period and participant races. Thirdly, hypoglycemia was the most frequent adverse event after MSCs treatment, which was associated with unadjusted exogenous insulin dosage. Finally, there were no treatment related serious adverse events suggesting that this therapeutic approach is safe.

The weaknesses of this meta-analysis arise from the potential biases in many of the trial reports, especially for complex interventions, which may have produced unreliable results. Although we used a random-effects model, the interpretation of the results of this meta-analysis requires some caution, given the high heterogeneity observed in the overall primary analysis. It is plausible to assume that this high heterogeneity likely reflects differences in the participant demographics, ethno-racial characteristics, interventions, length of follow-up, and in the choice of outcome indicators as the reasons for high heterogeneity. Besides, we consider the biggest limitation in the pooled analyses that we present here, both in the number and quality of trials. In view of the above, publication bias failed to be proceeded.

Notwithstanding, we have aimed to make full use of these clinical data, so as to suggest further study design and clinical application.

## Conclusions

This meta-analysis found evidence of beneficial effects in HbA1c levels of MSCs treatment for diabetes. MSCs therapy may be an effective and safe intervention in subjects with DM, especially T2DM and it can be an emerging and promising therapeutic modality for the treatment of diabetes. However, considerable uncertainty remains about which mechanism works, the duration of therapeutic effect, and who is most likely to benefit. Due to the limited studies, a number of high-quality as well as large-scale RCTs should be performed to confirm these conclusions.

## Supporting information

S1 Checklist PRISMA 2009 checklist. (DOC)
