# Supplementary material for: Clinical efficacy on glycemic control and safety of mesenchymal stem cells in patients with diabetes mellitus: Systematic review and meta-analysis of RCT data
Source: PLoS One. 2021 Mar 11;16(3):e0247662. doi: 10.1371/journal.pone.0247662 (PMC7951834; doi:10.1371/journal.pone.0247662)
Supplement: S1 Checklist — (DOC) [file pone.0247662.s001.doc]

| **Section/topic** | **#** | **Checklist item** | **Reported on page #** |
| --- | --- | --- | --- |
| **TITLE** | | |  |
| Title | 1 | Clinical Efficacy on Glycemic Control and Safety of Mesenchymal Stem Cells in Patients with Diabetes Mellitus: Systematic Review and Meta-analysis of RCT Data | 1 |
| **ABSTRACT** | | |  |
| Structured summary | 2 | Diabetes mellitus as a chronic metabolic disease is threatening human health seriously. No article has been published to summarize the efficacy and safety of mesenchymal stem cells (MSCs) in RCT trials (RCTs). The aim of this study was to systematically review the evidence from RCTs and to comprehensively assess the therapeutic efficacy and safety of MSCs in diabetes. Inclusion criteria included any age and sex of people diagnosed DM, without any additional complications, and the trials compared MSCs with placebo or MSCs as an adjunct treatment to insulin. We searched PubMed, Web of Science, Ovid, the Cochrane Library and CNKI databases using the following key words: (“Mesenchymal Stem Cells, Mesenchymal Stromal Cells, Wharton's Jelly Cells, progenitor cells, bone marrow” or “MSCs”) AND (“diabetes mellitus” or “DM”) AND (“English language” OR “Chinese language”). The retrieval time was from establishment of these databases to December 12, 2019. 7 RCTs were eligible for inclusion in the meta-analysis and were assessed for quality. we consider the biggest limitation in the pooled analyses that we present here, both in the number and quality of trials. MSCs therapy may be an effective and safe intervention in subjects with DM. No registration numbers. | 1-2 |
| **INTRODUCTION** | | |  |
| Rationale | 3 | Stem cell is a class of initial cells with self-renewing, high proliferation and multiple-directional differentiation ability in certain conditions. MSC is one of typical representatives of stem cells and attractive candidates for cell-based regenerative medicine with its multidirectional differentiation potential and immunomodulatory properties. Some studies MSCs infusion ameliorated hyperglycemia and increased serum C-peptide levels in DM via various approaches. | 2 |
| Objectives | 4 | Patients were selected with any age and sex of people diagnosed DM, without any additional complications, and the trials compared MSCs with placebo or MSCs as an adjunct treatment to insulin. | 2 |
| **METHODS** | | |  |
| Protocol and registration | 5 | None. | - |
| Eligibility criteria | 6 | RCTs and reported in English or Chinese. | 2 |
| Information sources | 7 | PubMed, Web of Science, Ovid, the Cochrane Library and CNKI databases were searched until Jan 4, 2020. | 3 |
| Search | 8 | Search (((diabetes[MeSH Terms]) AND ((((((Mesenchymal Stem Cell[MeSH Terms]) OR Mesenchymal Stromal Cell[MeSH Terms]) OR Bone Marrow Stromal Cell[MeSH Terms]) OR Bone Marrow derived stem Cell[MeSH Terms]) OR Mesenchymal Progenitor Cell[MeSH Terms]) OR Wharton's Jelly Cells[MeSH Terms]))) AND "english"[Language] in Pubmed. | 3 |
| Study selection | 9 | Two researchers independently screened literatures and extracted data (He and Kong). Divergences were arbitrated and resolved by a third reviewer. For all eligible studies, we extracted information. When reports lacked information, we sent e-mails to contact corresponding authors. The units were transformed and unified based on related specifications. If the data were inconsistent from main body to tables, refer to the former. | 3 |
| Data collection process | 10 | Two researchers independently extracted data from eligible studies by using information forms, finally compared and synthesized these data. | 3 |
| Data items | 11 | Included first author name, published year, settings, study design, population characteristics, interventions, outcome of interest, duration of follow-up, adverse events, and risk classification. | 3 |
| Risk of bias in individual studies | 12 | The Cochrane Risk of Bias tool was used to assess the quality of included studies. The five domains we assessed were: random sequence generation, allocation concealment, blind, incomplete outcome data and selective reporting. | 3 |
| Summary measures | 13 | We compared the MSC treatment groups from the selected trials with their respective control groups using Review Manager Version 5.3 software. The adverse effect of treatment was summarized using RR. | 3 |
| Synthesis of results | 14 | The I^2^ statistic were calculated to assess heterogeneity among these trials. I^2^＜50% was considered as a low level of heterogeneity, fixed-effects models were used; or random-effects models were used. The MSCs medicinal effects were reflected by the mean MD with 95% CI, as well as P＜0.05 was considered to be statistically significant. | 3 |

Page 1 of 2

| **Section/topic** | **#** | **Checklist item** | **Reported on page #** |
| --- | --- | --- | --- |
| Risk of bias across studies | 15 | The attrition bias caused by withdrawing that may affect the cumulative evidence. | 3 |
| Additional analyses | 16 | Subgroup analyses were pre-specified and proceeded. (Supplementary Figure 2) | 3 |
| **RESULTS** | | |  |
| Study selection | 17 | We initially retrieved 1,372 citations from selected databases and prior bibliographies. Of these, the majority were excluded. After 176 of full-text articles were assessed, 169 studies were excluded because they were non-human clinical trials, non-RCTs, and combining many complications with type 2 diabetes. Three studies were subsequently excluded because they used other stem cells simultaneously and did not provide available data. Finally, a total of 7 RCTs were eligible for inclusion in the meta-analysis and were assessed for quality. A flow diagram showing the selection process of studies is summarized in Supplementary Figure 1. | 4 |
| Study characteristics | 18 | The baseline characteristics of the included trials are presented in Table 1. | 4 |
| Risk of bias within studies | 19 | Presented in Supplementary Table 2. | 4 |
| Results of individual studies | 20 | Among 7 RCTs, 5 studies showed significant reduction of fasting plasma glucose (FPG) after MSCs therapy; 5 studies showed increased or decreased changes of hemoglobin (Hb) A1c after MSCs therapy; 5 studies provided data about fasting C-peptide, but did not get uniform conclusion. | 5 |
| Synthesis of results | 21 | Meta-analysis results showed that there were no significant differences in the reduction of fasting plasma glucose (FPG) compared to the baseline [random-effects, mean difference (MD) =-1.05, 95% confidence interval (CI) (-2.26,0.16), P<0.01, I^2^=94%] and the control group [MD=-0.62, 95%CI (-1.46,0.23), P<0.01, I^2^=87%]. The MSCs treatment group showed a significant decrease in hemoglobin (Hb) A1c [random-effects, MD=-1.32, 95%CI (-2.06, -0.57), P<0.01, I^2^=90%] after treatment. Additionally, HbA1c reduced more significantly in MSC treatment group than in control group [random-effects, MD=-0.87, 95%CI (-1.53, -0.22), P<0.01, I^2^=82%] at the end of follow-up. However, as for fasting C-peptide levels, the estimated pooled MD showed that there was no significant increase [MD=-0.07, 95%CI (-0.30, 0.16), P<0.01, I^2^=94%] in MSCs treatment group compared with that in control group. Notably, there was not significantly different in the incidence of adverse events between MSCs treatment group and control group [relative risk (RR)=0.98, 95%CI (0.72, 1.32), P=0.02, I^2^=70%]. The mostly observed adverse reaction in MSCs treatment group was hypoglycemia (29.95%). | 5 |
| Risk of bias across studies | 22 | The attrition bias caused by withdrawing that may affect the cumulative evidence. | 5 |
| Additional analysis | 23 | Subgroup analyses were pre-specified and proceeded. (Supplementary Figure 2) | 6 |
| **DISCUSSION** | | |  |
| Summary of evidence | 24 | Firstly, most of RCTs focus on T2DM, MSCs as add-on treatment to insulin can decrease HbA1c levels. Secondly, MSC-based cell treatment was associated with follow-up period and participant nationality. Thirdly, hypoglycemia was the most frequent adverse event after MSCs treatment. Although we used a random-effects model, the interpretation of the results of this meta-analysis requires some caution, given the high heterogeneity observed in the overall primary analysis. Notwithstanding, we have tried our best to make full use of these clinical data, so as to give out a hint to further study design and clinical application. | 6 |
| Limitations | 25 | The weaknesses of this meta-analysis arise from the potential biases in many of the trial reports, especially for complex interventions, which may have produced unreliable results. | 7 |
| Conclusions | 26 | MSCs therapy may be an effective and safe intervention in subjects with DM. | 8 |
| **FUNDING** | | |  |
| Funding | 27 | This work was supported by Natural Science Foundation of China (Grant No. 81801278), Natural Science Foundation of Hebei Province (Grant No. H2019206637), China Scholarship Council (Grant No. 201608130015), Hebei University Science and technology research project (Grant No. ZD2019049), Excellent Overseas researcher Program in Hebei Provincial Department of Human Resources and Social Security (Grant No. C20190509). | 8 |

*From:*  Moher D, Liberati A, Tetzlaff J, Altman DG, The PRISMA Group (2009). Preferred Reporting Items for Systematic Reviews and Meta-Analyses: The PRISMA Statement. PLoS Med 6(7): e1000097. doi:10.1371/journal.pmed1000097

For more information, visit: **www.prisma-statement.org**.

Page 2 of 2
